# Supplementary figures and images for: Deciphering the influence of evolutionary legacy and functional constraints on the patella: an example in modern rhinoceroses amongst perissodactyls
Source: PeerJ. 2024 Oct 25;12:e18067. doi: 10.7717/peerj.18067 (PMC11514768; doi:10.7717/peerj.18067)

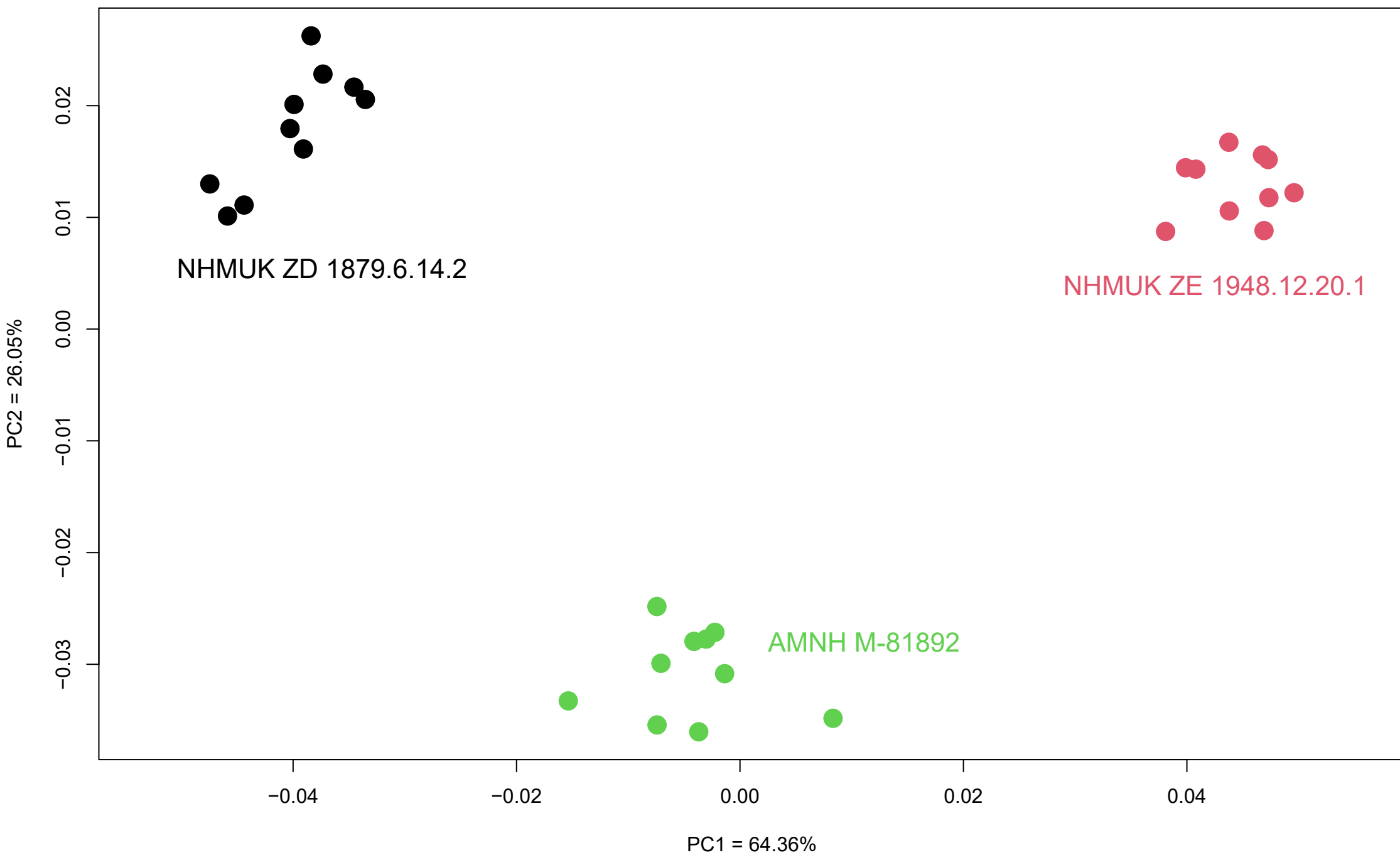

Supplement: Supplemental Information 1 — Each anatomical landmark configuration was digitized ten times on three specimens of Dicerorhinus sumatrensis chosen to display the fewer morphological difference as possible. Each colour corresponds to a specimen (green: AMNH M-81892, black: NHMUK ZD 1879.6.14.2, red: NHMUK ZE 1948.12.20.1). The inter-specimen variation is lower than the intra-specimen error due to differences between landmark digitization. We concluded to the relevance of our anatomical landmark configuration to describe shape variation within our sample. [file peerj-12-18067-s001.pdf]

**A**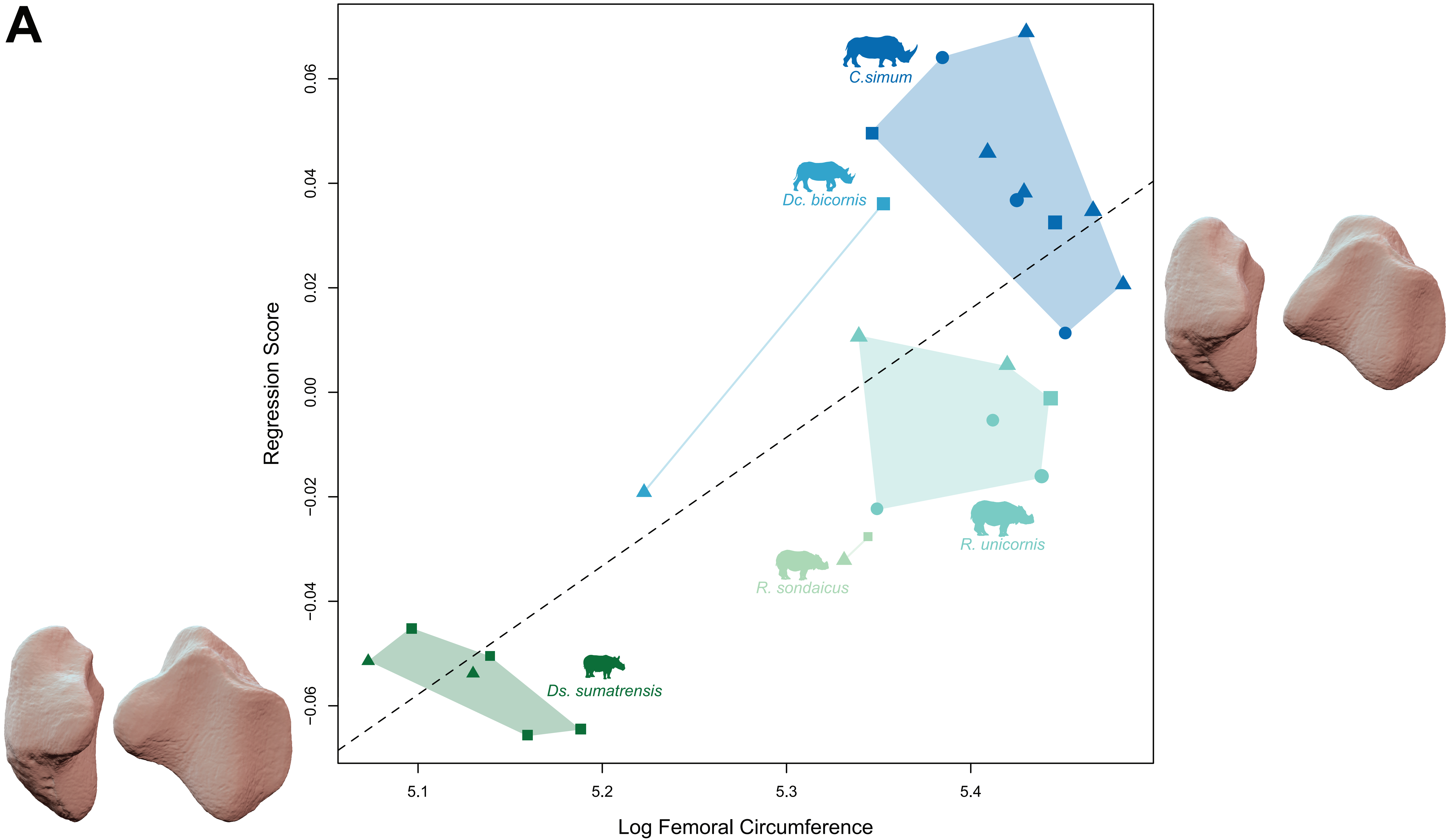**B**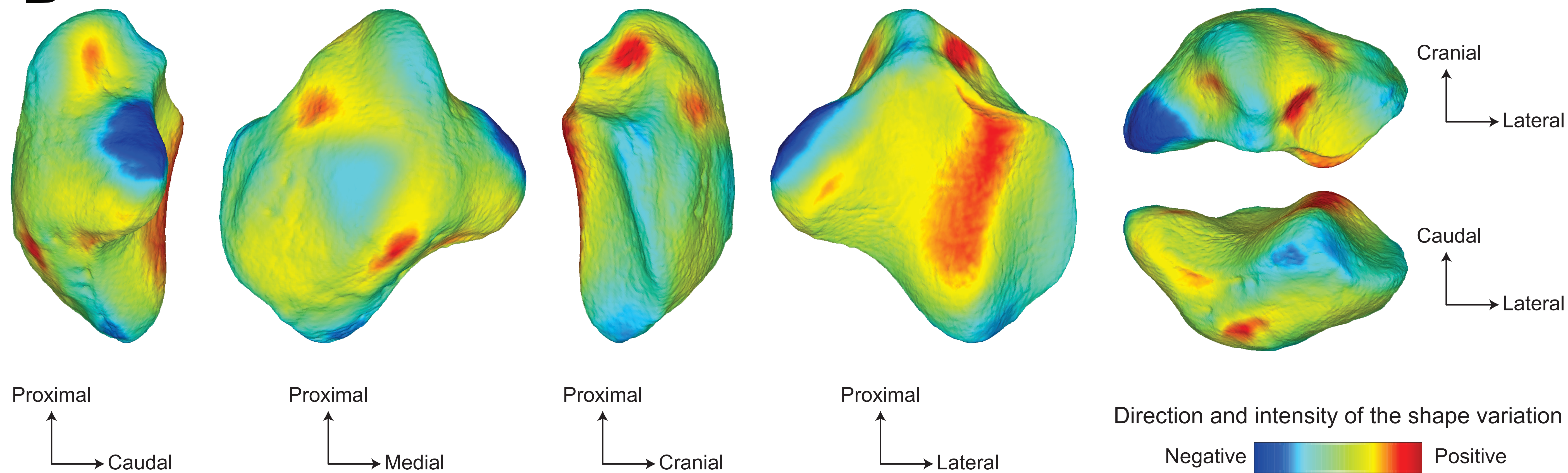

Supplement: Supplemental Information 2 — A: Regression plot with theoretical shapes associated with minimum and maximum fitted values (respectively in medial and caudal views). Colour code follows Figure 2. Point size is proportional to the log centroid size of each specimen. Symbols indicate the sex attribution (triangle: male; circle: female; square: unknown). Silhouettes of C. simum, Dc. bicornis, Ds. sumatrensis, R. sondaicus, and R. unicornis are personal creations. All other silhouettes provided by www.phylopic.org under the Creative Commons license. Tree generated by our R code provided as Supplemental Data. B: Colour maps of the location and intensity of the shape deformation. The shape associated with the maximal femoral circumference value of the Procrustes ANOVA was coloured depending on its distance to the shape associated with the minimal value. Green indicates no deformation; blue indicates a negative deformation of high intensity; red indicates a positive deformation of high intensity. Plot and theoretical 3D models generated by our R code provided as Supplemental Data (using the specimen Dicerorhinus sumatrensis AMNH M-81892 as a template for deformation of the meshes). [file peerj-12-18067-s002.pdf]

**A**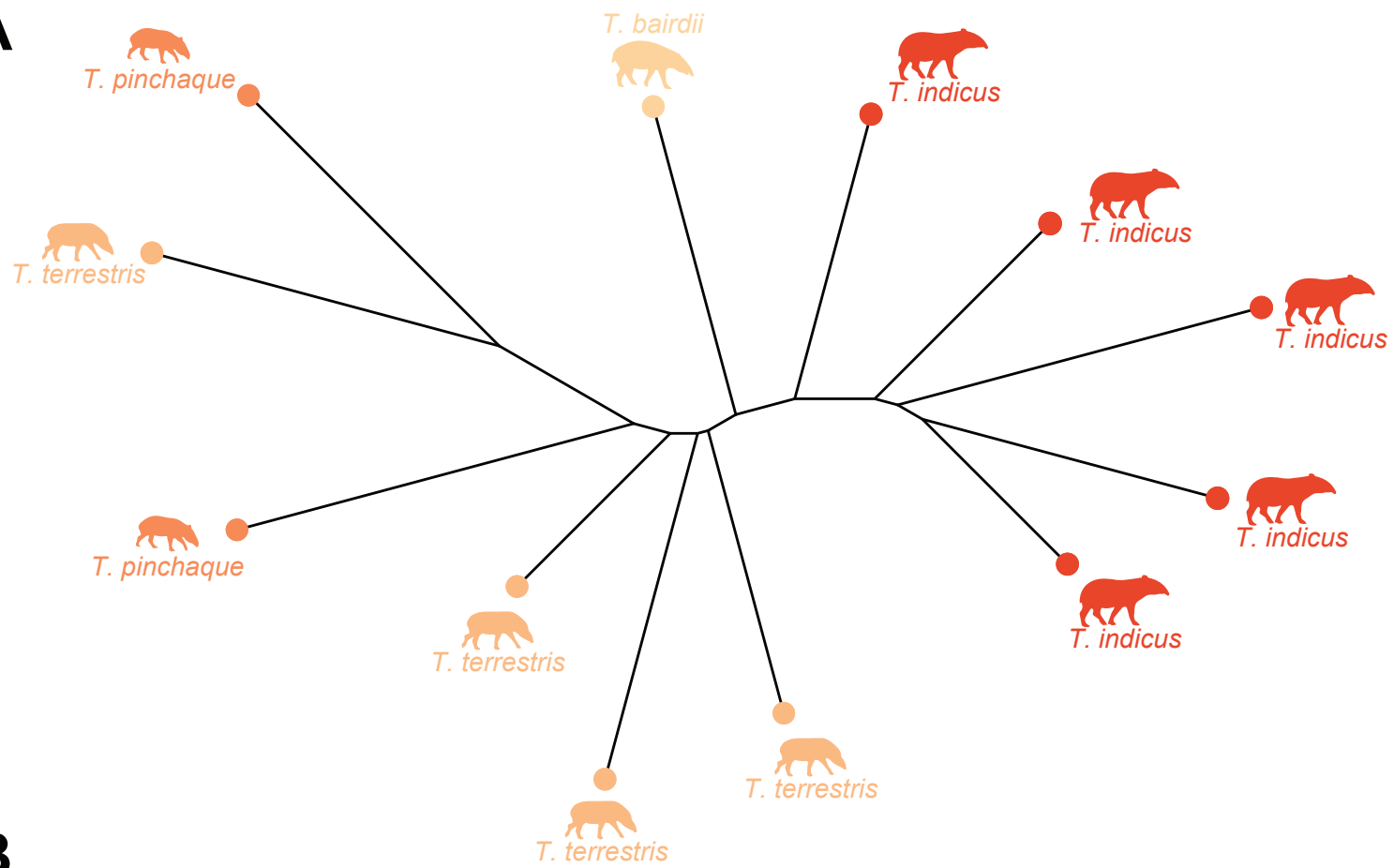**B**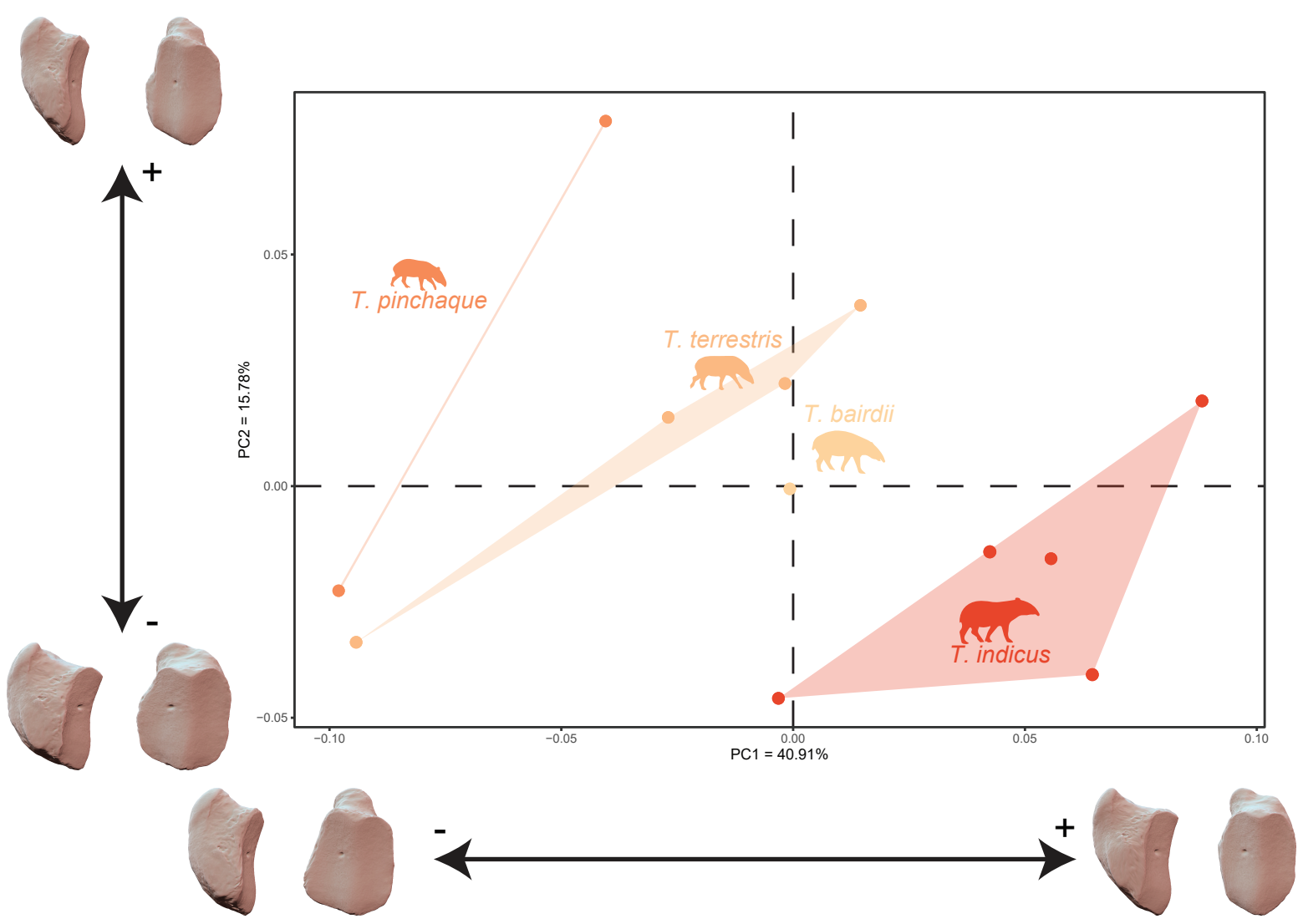

Supplement: Supplemental Information 3 — A: Neighbour Joining tree computed on all PC scores obtained from the PCA performed on shape data of tapirs only. Colour code follows Figure 2. Symbols indicate age class as in Table 1 (triangle: subadult; circle: adult). Point size is proportional to the log centroid size of each specimen. Silhouette of T. indicus is personal creation. All other silhouettes provided by www.phylopic.org under the Creative Commons license. Tree generated by our R code provided as Supplemental Data. B: Morphospace of the two first axes of the PCA performed on morphometric data of tapirs and minimal and maximal theoretical shape associated with this variation (respectively in medial and caudal views). Colour codes follow Figure 2. Symbols indicate age class as in Table 1 (triangle: subadult; circle: adult). Point size is proportional to the mean log centroid size of each specimen. T. indicus, the heaviest tapir and sister-group of all other three species, occupies highest values on PC1 and lowest on PC1. T. bairdii occupies null values for both axes. T. terrestris and T. pinchaque, the lightest species being sister-taxa together, occupy lowest PC1 values and highest PC2 values. Plot and theoretical 3D models generated by our R code provided as Supplemental Data (using the specimen Tapirus terrestris RBINS 1185D as a template for deformation of the meshes). [file peerj-12-18067-s003.pdf]

**A**

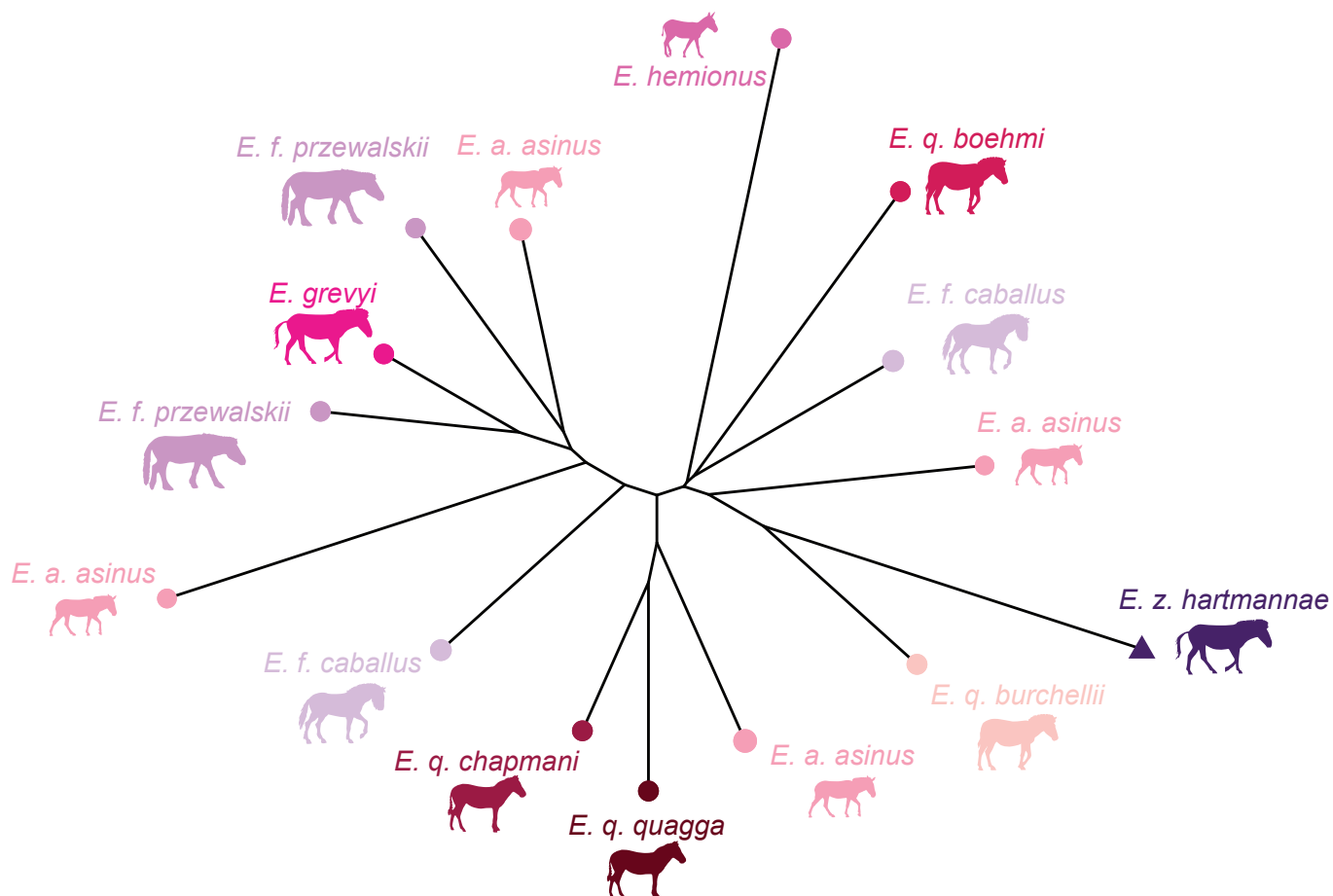

**B**

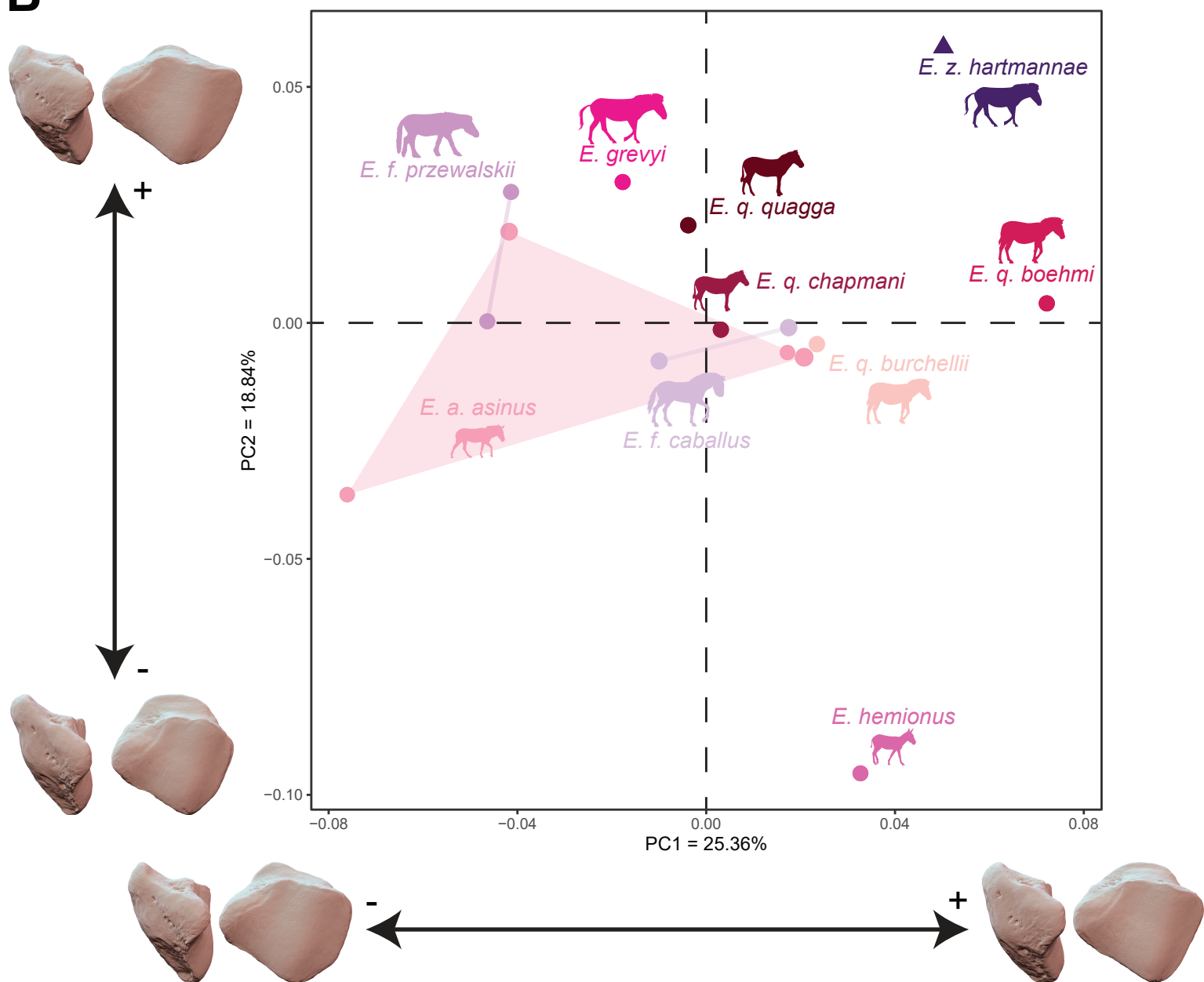

Supplement: Supplemental Information 4 — A: Neighbour Joining tree computed on all PC scores obtained from the PCA performed on shape data of equids only. Colour code follows Figure 2. Symbols indicate age class as in Table 1 (triangle: subadult; circle: adult). Point size is proportional to the log centroid size of each specimen. Silhouettes of E. z. hartmannae and E. grevyi are personal creations. All other silhouettes provided by www.phylopic.org under the Creative Commons license. Tree generated by our R code provided as Supplemental Data. B: Morphospace of the two first axes of the PCA performed on morphometric data of equids and minimal and maximal theoretical shape associated with this variation (respectively in medial and caudal views). Colour codes follow Figure 2. Symbols indicate age class as in Table 1 (triangle: subadult; circle: adult). Point size is proportional to the mean log centroid size of each specimen. Most zebras occupy highest PC1 and PC2 values but remain mixed with some donkeys and horses. E. hemionus is isolated towards the lowest PC2 values. Donkeys and horses occupy mostly null and negative PC1 values. Plot and theoretical 3D models generated by our R code provided as Supplemental Data (using the specimen Equus quagga chapmani RBINS 1218 as a template for deformation of the meshes). [file peerj-12-18067-s004.pdf]

**A**

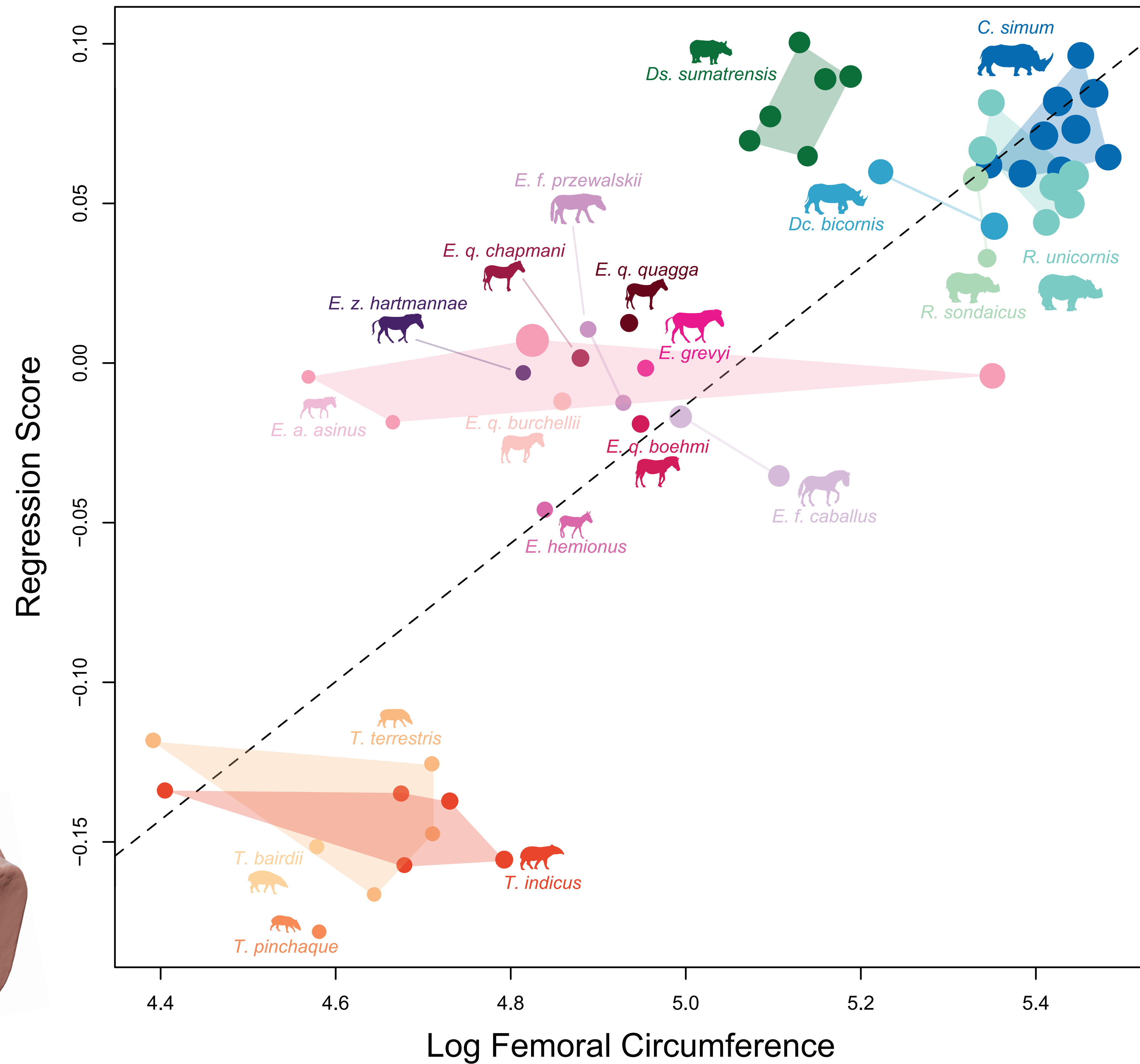

**B**

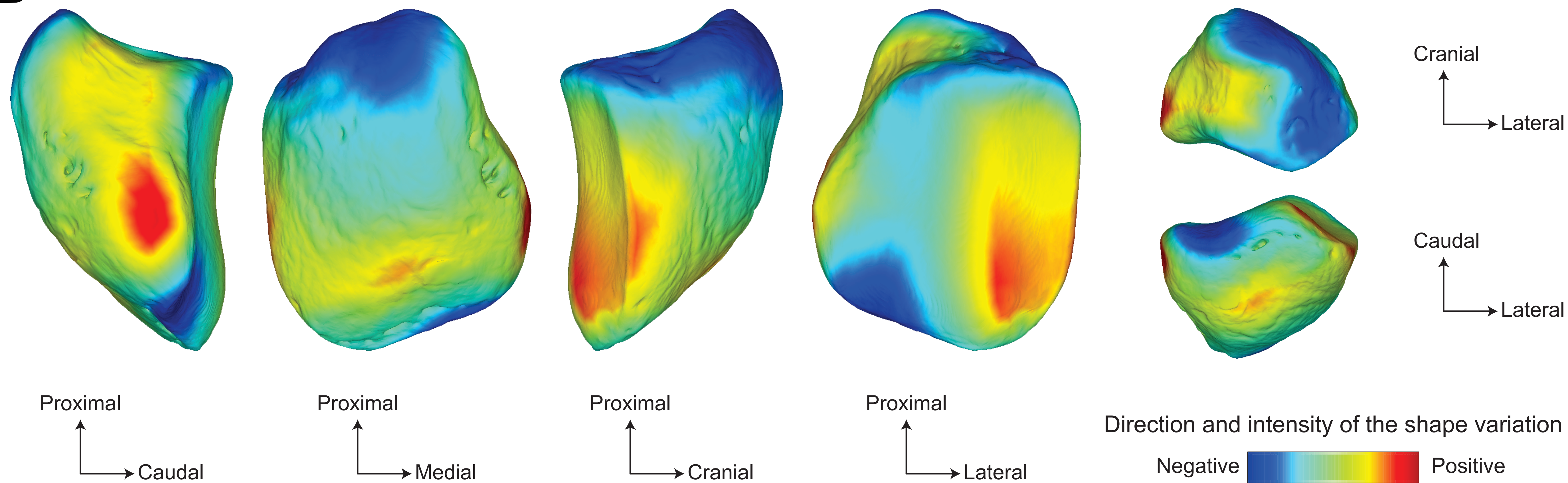

Supplement: Supplemental Information 6 — A: Regression plot with theoretical shapes associated with minimum and maximum fitted values (respectively in medial and caudal views). Colour code follows Figure 2. Point size is proportional to the log centroid size of each specimen. Silhouettes of C. simum, Dc. bicornis, Ds. sumatrensis, E. z. hartmannae, E. grevyi, R. sondaicus, R. unicornis and T. indicus are personal creations. All other silhouettes provided by www.phylopic.org under the Creative Commons license. B: Colour maps of the location and intensity of the shape deformation. The shape associated with the maximal femoral circumference value of the Procrustes ANOVA was coloured depending on its distance to the shape associated with the minimal value. Green indicates no deformation; blue indicates a negative deformation of high intensity; red indicates a positive deformation of high intensity. Plot and theoretical 3D models generated by our R code provided as Supplemental Data (using the specimen Diceros bicornis NHMUK ZD 1879.9.26.6 as a template for deformation of the meshes). [file peerj-12-18067-s006.pdf]
