# Supplementary material for: Deciphering the influence of evolutionary legacy and functional constraints on the patella: an example in modern rhinoceroses amongst perissodactyls
Source: PeerJ. 2024 Oct 25;12:e18067. doi: 10.7717/peerj.18067 (PMC11514768; doi:10.7717/peerj.18067)
Supplement: Supplemental Information 5 — Shapes are computed as the mean conformations of all specimens per species. For species represented by a single specimen, shapes correspond to the specimen.Colour code follows Figure 2. Silhouettes of C. simum, Dc. bicornis, Ds. sumatrensis, E. z. hartmannae, E. grevyi, R. sondaicus, R. unicornis and T. indicus are personal creations. All other silhouettes provided by www.phylopic.org under the Creative Commons license. Theoretical 3D models generated by our R code provided as Supplemental Data (using the specimen Diceros bicornis NHMUK ZD 1879.9.26.6 as a template for deformation of the meshes). [file peerj-12-18067-s005.pdf]

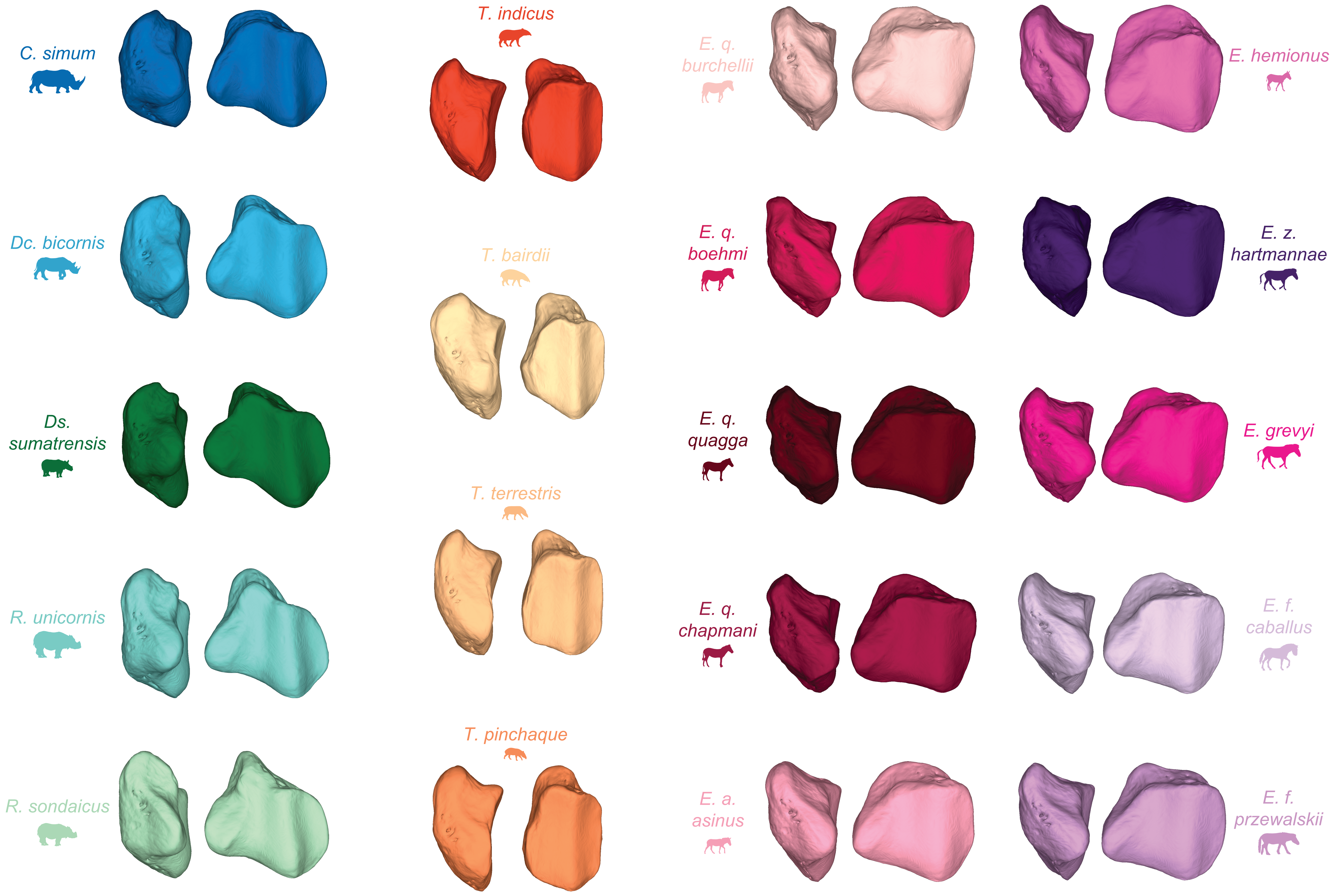

C. simum
T. indicus
E. q. burchellii
E. hemionus
Dc. bicornis
T. bairdii
E. q. boehmi
E. z. hartmannae
Ds. sumatrensis
E. q. quagga
E. grevyi
T. terrestris
R. unicornis
E. q. chapmani
E. f. caballus
T. pinchaque
R. sondaicus
E. a. asinus
E. f. przewalskii
