# Supplementary material for: Deciphering the influence of evolutionary legacy and functional constraints on the patella: an example in modern rhinoceroses amongst perissodactyls
Source: PeerJ. 2024 Oct 25;12:e18067. doi: 10.7717/peerj.18067 (PMC11514768; doi:10.7717/peerj.18067)
Supplement: Supplemental Information 7 [file peerj-12-18067-s007.docx]

| **Taxon** | **Institution** | **Collection number** | **3D acquisition** | **MorphoSource ARK Identifier** | **DOI** |
| --- | --- | --- | --- | --- | --- |
| *Ceratotherium simum* | AMNH | M-51854 | SL | <http://n2t.net/ark:/87602/m4/606880> | <https://doi.org/10.17602/M2/M606880> |
| *Ceratotherium simum* | AMNH | M-51855 | SL | <http://n2t.net/ark:/87602/m4/606884> | <https://doi.org/10.17602/M2/M606884> |
| *Ceratotherium simum* | AMNH | M-51857 | SL | <http://n2t.net/ark:/87602/m4/606888> | <https://doi.org/10.17602/M2/M606888> |
| *Ceratotherium simum* | AMNH | M-51858 | SL | <http://n2t.net/ark:/87602/m4/606892> | <https://doi.org/10.17602/M2/M606892> |
| *Ceratotherium simum* | AMNH | M-81815 | SL | <http://n2t.net/ark:/87602/m4/606896> | <https://doi.org/10.17602/M2/M606896> |
| *Ceratotherium simum* | BICPC | NH.CON.37 | SL | <http://n2t.net/ark:/87602/m4/606904> | <https://doi.org/10.17602/M2/M606904> |
| *Ceratotherium simum* | BICPC | NH.CON.110 | SL | <http://n2t.net/ark:/87602/m4/606913> | <https://doi.org/10.17602/M2/M606913> |
| *Ceratotherium simum* | BICPC | NH.CON.112 | SL | <http://n2t.net/ark:/87602/m4/606917> | <https://doi.org/10.17602/M2/M606917> |
| *Ceratotherium simum* | MNHN | ZM-MO-2005-297 | CT | <http://n2t.net/ark:/87602/m4/607110> | <https://doi.org/10.17602/M2/M607110> |
| *Ceratotherium simum* | NHMUK | ZD 2018.143 | SL | <http://n2t.net/ark:/87602/m4/606956> | <https://doi.org/10.17602/M2/M606956> |
| *Diceros bicornis* | AMNH | M-113777 | SL | <http://n2t.net/ark:/87602/m4/606933> | <https://doi.org/10.17602/M2/M606933> |
| *Diceros bicornis* | MNHN | ZM-AC-1944-278 | CT | <http://n2t.net/ark:/87602/m4/606925> | <https://doi.org/10.17602/M2/M606925> |
| *Diceros bicornis* | NHMUK | ZD 1879.9.26.6 | CT | <http://n2t.net/ark:/87602/m4/607115> | <https://doi.org/10.17602/M2/M607115> |
| *Dicerorhinus sumatrensis* | AMNH | M-81892 | SL | <http://n2t.net/ark:/87602/m4/606938> | <https://doi.org/10.17602/M2/M606938> |
| *Dicerorhinus sumatrensis* | NHMUK | ZD 1879.6.14.2 | SL | <http://n2t.net/ark:/87602/m4/606961> | <https://doi.org/10.17602/M2/M606961> |
| *Dicerorhinus sumatrensis* | NHMUK | ZD 1894.9.24.1 | SL | <http://n2t.net/ark:/87602/m4/607044> | <https://doi.org/10.17602/M2/M607044> |
| *Dicerorhinus sumatrensis* | NHMUK | ZE 1948.12.20.1 | SL | <http://n2t.net/ark:/87602/m4/607054> | <https://doi.org/10.17602/M2/M607054> |
| *Dicerorhinus sumatrensis* | NHMUK | ZE 1949.1.11.1 | SL | <http://n2t.net/ark:/87602/m4/607063> | <https://doi.org/10.17602/M2/M607063> |
| *Dicerorhinus sumatrensis* | NHMUK | ZD 2004.23 | SL | <http://n2t.net/ark:/87602/m4/606966> | <https://doi.org/10.17602/M2/M606966> |
| *Rhinoceros sondaicus* | MNHN | ZM-AC-A7970 | SL | <http://n2t.net/ark:/87602/m4/606970> | <https://doi.org/10.17602/M2/M606970> |
| *Rhinoceros sondaicus* | NHMUK | ZD 1871.12.29.7 | SL | <http://n2t.net/ark:/87602/m4/607071> | <https://doi.org/10.17602/M2/M607071> |
| *Rhinoceros unicornis* | AMNH | M-35759 | SL | <http://n2t.net/ark:/87602/m4/606942> | <https://doi.org/10.17602/M2/M606942> |
| *Rhinoceros unicornis* | AMNH | M-54454 | SL | <http://n2t.net/ark:/87602/m4/606951> | <https://doi.org/10.17602/M2/M606951> |
| *Rhinoceros unicornis* | MNHN | ZM-AC-1967-101 | SL | <http://n2t.net/ark:/87602/m4/607105> | <https://doi.org/10.17602/M2/M607105> |
| *Rhinoceros unicornis* | NHMUK | ZE 1961.5.10.1 | SL | <http://n2t.net/ark:/87602/m4/607091> | <https://doi.org/10.17602/M2/M607091> |
| *Rhinoceros unicornis* | NHMUK | ZD 1972.822 | SL | <http://n2t.net/ark:/87602/m4/607096> | <https://doi.org/10.17602/M2/M607096> |
| *Rhinoceros unicornis* | NHMUK | ZD 1884.12.1.2 | SL | <http://n2t.net/ark:/87602/m4/607101> | <https://doi.org/10.17602/M2/M607101> |
| *Tapirus bairdii* | MVZ | 141172 | LS | <http://n2t.net/ark:/87602/m4/M169313> | <https://doi.org/10.17602/M2/M169313> |
| *Tapirus indicus* | MNHN | ZM-AC-1931-528 | CT | <http://n2t.net/ark:/87602/m4/607124> | <https://doi.org/10.17602/M2/M607124> |
| *Tapirus indicus* | MNHN | ZM-AC-1935-460 | CT | <http://n2t.net/ark:/87602/m4/607129> | <https://doi.org/10.17602/M2/M607129> |
| *Tapirus indicus* | NMB | 8125 | CT | <http://n2t.net/ark:/87602/m4/607135> | <https://doi.org/10.17602/M2/M607135> |
| *Tapirus indicus* | RBINS | 1184D | LS | <http://n2t.net/ark:/87602/m4/606976> | <https://doi.org/10.17602/M2/M606976> |
| *Tapirus indicus* | RBINS | 1184E | LS | <http://n2t.net/ark:/87602/m4/606981> | <https://doi.org/10.17602/M2/M606981> |
| *Tapirus pinchaque* | MNHN | ZM-AC-1982-34 | CT | <http://n2t.net/ark:/87602/m4/607137> | <https://doi.org/10.17602/M2/M607137> |
| *Tapirus pinchaque* | MNHN | ZM-AC-1877-765 | CT | <http://n2t.net/ark:/87602/m4/607151> | <https://doi.org/10.17602/M2/M607151> |
| *Tapirus terrestris* | MNHN | ZM-AC-1937-1 | CT | <http://n2t.net/ark:/87602/m4/607152> | <https://doi.org/10.17602/M2/M607152> |
| *Tapirus terrestris* | MNHN | ZM-MO-1990-20 | CT | <http://n2t.net/ark:/87602/m4/607160> | <https://doi.org/10.17602/M2/M607160> |
| *Tapirus terrestris* | RBINS | 1185D | LS | <http://n2t.net/ark:/87602/m4/606987> | <https://doi.org/10.17602/M2/M606987> |
| *Tapirus terrestris* | RBINS | 1185E | LS | <http://n2t.net/ark:/87602/m4/606992> | <https://doi.org/10.17602/M2/M606992> |
| *Equus africanus asinus* | MNHN | ZM-AC-1893-634 | CT | <http://n2t.net/ark:/87602/m4/607161> | <https://doi.org/10.17602/M2/M607161> |
| *Equus africanus asinus* | MNHN | ZM-2005-717 | CT | <http://n2t.net/ark:/87602/m4/607174> | <https://doi.org/10.17602/M2/M607174> |
| *Equus africanus asinus* | RBINS | 12970 | LS | <http://n2t.net/ark:/87602/m4/606999> | <https://doi.org/10.17602/M2/M606999> |
| *Equus africanus asinus* | RBINS | 13076 | LS | <http://n2t.net/ark:/87602/m4/607003> | <https://doi.org/10.17602/M2/M607003> |
| *Equus hemionus* | MNHN | ZM-AC-1880-1103 | CT | <http://n2t.net/ark:/87602/m4/607184> | <https://doi.org/10.17602/M2/M607184> |
| *Equus ferus caballus* | MNHN | ZM-AC-A541 | CT | <http://n2t.net/ark:/87602/m4/607188> | <https://doi.org/10.17602/M2/M607188> |
| *Equus ferus caballus* | MVZ | 162289 | LS | <http://n2t.net/ark:/87602/m4/M122840> | <https://doi.org/10.17602/M2/M122840> |
| *Equus ferus przewalskii* | MNHN | ZM-AC-1975-124 | CT | <http://n2t.net/ark:/87602/m4/607196> | <https://doi.org/10.17602/M2/M607196> |
| *Equus ferus przewalskii* | RBINS | 14281 | LS | <http://n2t.net/ark:/87602/m4/607008> | <https://doi.org/10.17602/M2/M607008> |
| *Equus burchellii granti* | RBINS | 33386 | LS | <http://n2t.net/ark:/87602/m4/607013> | <https://doi.org/10.17602/M2/M607013> |
| *Equus grevyi* | RBINS | 32166 | LS | <http://n2t.net/ark:/87602/m4/607019> | <https://doi.org/10.17602/M2/M607019> |
| *Equus quagga boehmi* | RBINS | 12129 | LS | <http://n2t.net/ark:/87602/m4/607024> | <https://doi.org/10.17602/M2/M607024> |
| *Equus quagga chapmani* | RBINS | 1218 | LS | <http://n2t.net/ark:/87602/m4/607029> | <https://doi.org/10.17602/M2/M607029> |
| *Equus quagga quagga* | IMNH | R2425 | LS | <http://n2t.net/ark:/87602/m4/M121755> | <https://doi.org/10.17602/M2/M121755> |
| *Equus zebra hartmannae* | RBINS | 3974 | LS | <http://n2t.net/ark:/87602/m4/607034> | <https://doi.org/10.17602/M2/M607034> |

LS: laser scanner; SL: structured light surface scanner; P: photogrammetry; CT: CTscan.
